# BSMAP: whole genome bisulfite sequence MAPping program

**DOI:** 10.1186/1471-2105-10-232

**Published:** 2009-07-27

**Authors:** Yuanxin Xi, Wei Li

**Affiliations:** 1 Division of Biostatistics, Dan L Duncan Cancer Center, Department of Molecular and Cellular Biology, Baylor College of Medicine, One Baylor Plaza, Houston, TX 77030, USA

## Abstract

**Background:**

Bisulfite sequencing is a powerful technique to study DNA cytosine methylation. Bisulfite treatment followed by PCR amplification specifically converts unmethylated cytosines to thymine. Coupled with next generation sequencing technology, it is able to detect the methylation status of every cytosine in the genome. However, mapping high-throughput bisulfite reads to the reference genome remains a great challenge due to the increased searching space, reduced complexity of bisulfite sequence, asymmetric cytosine to thymine alignments, and multiple CpG heterogeneous methylation.

**Results:**

We developed an efficient bisulfite reads mapping algorithm BSMAP to address the above issues. BSMAP combines genome hashing and bitwise masking to achieve fast and accurate bisulfite mapping. Compared with existing bisulfite mapping approaches, BSMAP is faster, more sensitive and more flexible.

**Conclusion:**

BSMAP is the first general-purpose bisulfite mapping software. It is able to map high-throughput bisulfite reads at whole genome level with feasible memory and CPU usage. It is freely available under GPL v3 license at .

## Background

**Figure 1:**
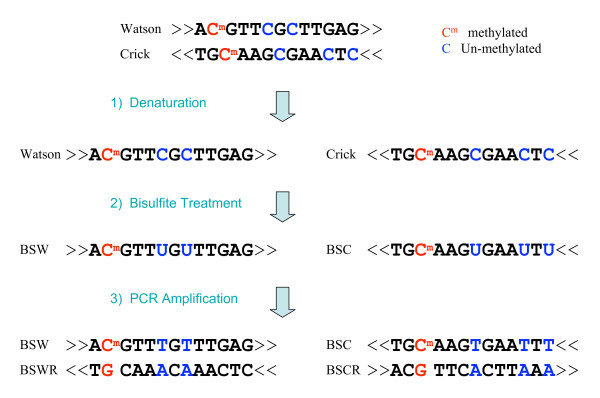
**Pipeline of bisulfite sequencing**. 1) Denaturation: separating Watson and Crick strands; 2) Bisulfite treatment: converting un-methylated cytosines (blue) to uracils; methylated cytosines (red) remain unchanged; 3) PCR amplification of bisulfite-treated sequences resulting in four distinct strands: Bisulfite Watson (BSW), bisulfite Crick (BSC), reverse complement of BSW (BSWR), and reverse complement of BSC (BSCR).

Cytosine (C) DNA methylation plays a crucial role in various biological processes such as gene expression, chromatin accessibility, and imprinting, as well as in many diseases including cancer. Over the decades, bisulfite sequencing [1] has remained the gold standard for DNA methylation analysis. Bisulfite treatment of DNA followed by PCR amplification leads to a chemical conversion of unmethylated Cs to Ts without affecting As, Gs, Ts or methylated Cs. This C to T conversion results in non-complementarity in the two strands of DNA (Figure 1). Following strand-specific and locus-specific PCR amplification, direct- or pyro-sequencing is used to determine the methylation ratio of any given C locus as the proportion of remaining Cs in all the sequencing reads. This PCR-based procedure is very labor intensive and time-consuming, and therefore inappropriate for high throughput studies.

Tremendous progress has been achieved in the past two years in the development of massively parallel sequencing such as Illumina/Solexa and Applied Biosystems/SOLiD. Tens of millions of short tags (36–75 bases) can now be simultaneously sequenced at less than 1% of the cost of traditional Sanger methods. Without the locus-specific PCR step, bisulfite treatment coupled with next-generation shotgun sequencing (BS-seq) [2-4] has become a powerful technique with the potential to quantitatively detect the methylation pattern of every C in the genome. Nevertheless, the mapping of millions of bisulfite reads to the reference genome remains a computational challenge.

### Problems

First, the searching space is significantly increased relative to the original reference sequence. Unlike normal sequencing, the Watson and Crick strands of bisulfite-treated sequences are not complementary to each other because the bisulfite conversion only occurs on Cs. As a result, there will be four distinct strands after PCR amplification: BSW (bisulfite Watson), BSWR (reverse complement of BSW), BSC (bisulfite Crick), and BSCR (reverse complement of BSC) (Figure 1). During shotgun sequencing, a bisulfite read is almost equally likely to be derived from any of the four strands.

Second, sequence complexity is reduced. In the mammalian genome, although ~19% of the bases are Cs and another 19% are Gs, only ~1.8% of dinucleotides are CpG dinucleotides. Because C methylation occurs almost exclusively at CpG dinucleotide, the vast majority of Cs in BSW and BSC strands will be converted to Ts. Therefore, most reads from the above two strands will be C-poor. However, PCR amplification will transcribe all Gs as Cs in BSWR and BSCR strands, so reads from those two strands are typically G-poor and have a normal C content. As a result, we expect the overall C content of bisulfite reads to be reduced by ~50%.

**Figure 2:**
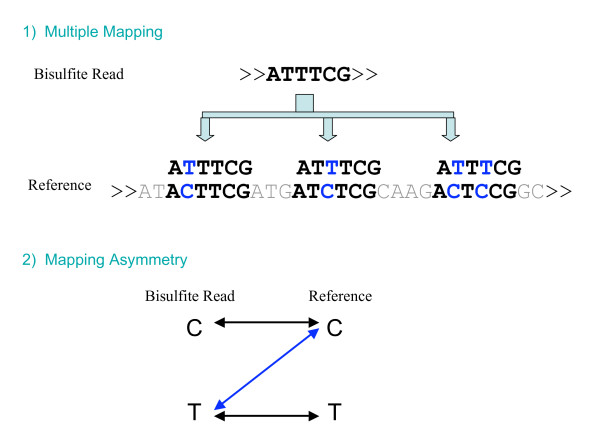
**Mapping of bisulfite reads**. 1) Increased search space due to the cytosine-thymine conversion in the bisulfite treatment. 2) Mapping asymmetry: thymines in bisulfite reads can be aligned with cytosines in the reference (illustrated in blue) but not the reverse.

Third, C to T mapping is asymmetric. The T in the bisulfite reads could be mapped to either C or T in the reference but not vice versa. This phenomenon not only increases the search space for mapping but also complicates the matching process (Figure 2). Efficient implementation of such asymmetric C/T matching is critical for mapping high-throughput bisulfite reads to the reference genome and is still lacking in current short read alignment software.

A common approach to overcome these issues is to convert all Cs to Ts and map the converted reads to the converted reference; then, the alignment results are post-processed to count false-positive bisulfite C/T alignments as mismatches, where a C in the BS-read is aligned to a T in the reference [2]. Although this all-inclusive C/T conversion is effective for reads derived from the C-poor strands, it is not appropriate for reads derived from the G-poor strands, where all the Cs are actually transcribed from Gs by PCR amplification and thus could not be converted to Ts during bisulfite treatment. During shotgun sequencing, however, a bisulfite read is almost equally likely to be derived from either the C-poor or the G-poor strands. There is no precise way to determine the original strand a bisulfite read is derived from. Furthermore, by ignoring the C/T mapping asymmetry, this strategy generates a large number of false-positive bisulfite mappings and greatly increases the computational load in a quadratic manner with an increase in the size of the reference sequence. In order to accurately extract the true bisulfite mappings in the post-processing stage, all mapping locations have to be recorded, even the non-unique mappings. Therefore, this approach is only practical for small reference sequences, where only the C-poor strands are sequenced. For example, Meissner et al. used this mapping strategy for reduced representation bisulfite sequencing (RRBS) [2], where the genomic DNA was digested by the Mspl restriction enzyme and 40–220 bp segments were selected for sequencing. The reference sequence (~27 M nt) is only about 1% of the whole mouse genome, covering 4.8% of the total CpG dinucleotides.

Lister et al. used another bisulfite mapping strategy in their study of DNA methylation in *Arabidopsis *[3]. Bisulfite reads were aligned to 3 reference sequences, namely the original genome and the two bisulfite-converted genomes with Cs converted to Ts for the forward strand and Gs converted to As for the reverse strand. The mapping results for all three references were combined to generate methylation information. Unlike the strategy used by Meissner et al. [2], this approach does not change the bisulfite read but rather captures possible bisulfite C/T alignments by allowing them to be mismatches. The biggest drawback of this approach is that the number of bisulfite C/T alignments that can be detected in a read is bounded by the number of mismatches allowed by the mapping software; this number might be further reduced by true mismatches such as SNPs. In this study, the bisulfite read length was 32 bp and 2 mismatches were allowed, so reads with more than 2 bisulfite C/T alignments, most of which were derived from CpG islands, were not detectable. This strategy could substantially compromise the mapping sensitivity.

Naturally, it is desirable to map bisulfite reads directly to the reference sequence. Cokus et al. [4] used a custom-made tool, CokusAlignment, to map bisulfite reads to the *Arabidopsis *genome. This method is based on a tree searching algorithm, which is both computationally intensive and memory demanding. CokusAlignment runs at a moderate speed (~25 reads/sec/CPU) with a relatively small genome (~120 M nt) by applying many project-specific optimizations, which might not be applicable to larger genomes or longer bisulfite reads. In addition, CokusAlignment does not support basic alignment functions such as gapped or pair-end alignment. From a practical point of view, this method is not suitable for general purpose bisulfite sequence mapping due to its slow speed, lack of functions, and excessive hardware requirements, especially for large genomes. To our knowledge, an efficient, multifunctional, general-purpose bisulfite sequence mapping software is not yet available, and the lack of such a tool has become a major bottleneck for whole-genome DNA methylation profiling using bisulfite sequencing.

**Figure 3:**
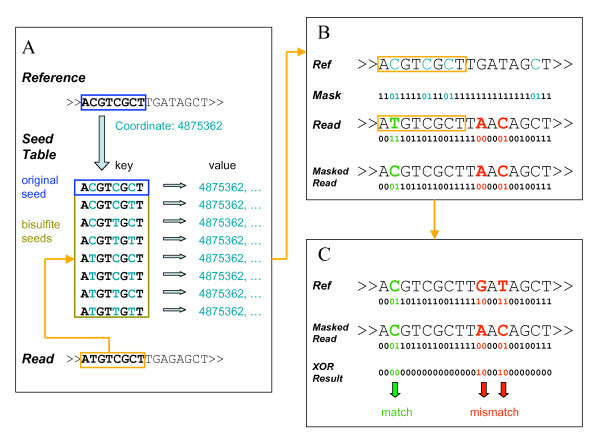
**BSMAP algorithm**. A) Bisulfite seed table, using the original seed and bisulfite variants as keys and corresponding coordinates in the reference genome as values. Each read was looked up in the seed table for potential mapping positions. B) A positional specific mask of the corresponding reference sequence was generated by setting 01 to C(light blue) and 11 to A, G, T(black). The original read was masked by a bitwise AND operation with the positional specific mask. C) The reference sequence and the masked read were compared with a bitwise XOR operation. Non-zero XOR results were counted as mismatches (red). Bisulfite alignment is marked in green.

We present here a general-purpose Bisulfite Sequence MAPping program, BSMAP, which addresses all the above issues. We used the general premise that all the C positions in the genome, where the asymmetric C/T transition can occur, are already known and can be used to guide the mapping of bisulfite reads. BSMAP masks Ts in the bisulfite reads as Cs (i.e., reverse bisulfite conversion) only at C positions in the original reference while keeping all other Ts in the bisulfite reads unchanged. BSMAP then maps the masked BS read directly to the reference. The asymmetric C/T conversion is achieved through position-specific bitwise masking of the bisulfite reads; this method is extremely fast. In addition, BSMAP is based on the more efficient HASH table seeding algorithm, which indexes the reference for all possible k-mers, called seeds, and only searches the locations where the seeds are perfectly matched with part of the read. By looking up the seed table, the majority of non-mapping positions are skipped, and the searching efficiency is greatly improved. The seeding length and patterns can also be adjusted to allow for a different number of mismatches. Because of these advantages, the seeding algorithm has been incorporated into most short read mapping software, including SOAP [5], ELAND, MAQ [6], and RMAP [7]. By combining fast seeding and bitwise masking (Figure 3), BSMAP offers a great improvement in performance as well as flexibility over the existing bisulfite mapping approaches.

## Results and discussion

### HASH table seeding

We implemented BSMAP based on the open source software SOAP (Short Oligonucleotide Alignment Program) [5]. To find all possible mapping positions, each bisulfite read is divided into 4 parts, two of which are combined to form 6 possible seeds. To find the possible mapping positions, these seeds are then looked up in a HASH table that is pre-compiled from the reference sequence. To accommodate the C/T mapping issue, the HASH table includes all possible seeds in the reference sequence with their bisulfite variants as keys and the corresponding coordinates as values. The bisulfite variants of a specific seed are generated by enumerating all possible C=>T combinations (Figure 3A).

### Bitwise masking

For each possible mapping location, the actual number of mismatches between the bisulfite read and the reference needs to be counted, allowing a T in the bisulfite read to map to a C in the reference. Each DNA nucleotide is represented by two bits (i.e., A: 00, C: 01, G:10, T:11) and DNA sequences are represented as binary strings. We transfer a T in the bisulfite read to C where a corresponding C in the reference by applying a bit mask. Specifically, a bitwise AND mask (01) is used to convert a T (11) to C (01) or keep a C (01) unchanged where the reference is a C (i.e., active); an AND mask (11), which actually does not change anything (i.e., inactive), is used where the reference is an A, G, or T (Figure 3B). The complete matching results between the original bisulfite reads and references are listed in Table 1.

**Table 1:** Bisulfite matching table after bitwise masking

Reference		A (00)	C (01)	G (10)	T (11)
Mask		11 (inactive)	01 (active)	11 (inactive)	11 (inactive)

Bisulfite Read	A(00)	00 (match)	01	10	11
	C(01)	10	00 (match)	11	10
	G(10) => A(00) if reference is C	10	01	00 (match)	01
	T(11) => C(01) if reference is C	11	00 (match)	01	00 (match)

The bitwise masks are also stored in a HASH table structure as values, with the corresponding sequences as keys. Counting bisulfite mismatches only adds one table query and one bitwise AND operation to a normal sequence mapping. Both operations can be executed very efficiently, and the computational load is only slightly increased.

### Mismatch counting

Mismatch counting is implemented through a bitwise exclusive OR (XOR) operation between the masked bisulfite read and the reference. For any two nucleotides, the XOR operation returns a zero if they are the same (match) and a non-zero (mismatch) otherwise. The number of mismatches between two sequences is the total number of non-zero two-bit segments. As illustrated in Figure 3B and 3C, the mask "01" generated by a C in the 2^nd ^position in the reference genome (light blue) converts the T in the original read to a C in the masked read (green); the result is a match (XOR result "00") in the mismatch counting. The T in the read is correctly aligned with the C in the reference without being identified as a mismatch. On the other hand, normal mismatches are not affected by the bisulfite mask, as indicated by the mismatches detected at the 10^th ^and 12^th ^nucleotides (red). In the 12^th ^nt position, a T in the read was identified as a mismatch to the C in the reference because the mask "11" corresponding to the T in the reference did not change the C in the read, illustrating the asymmetry of C/T alignment.

### Other BSMAP features

**Figure 4:**
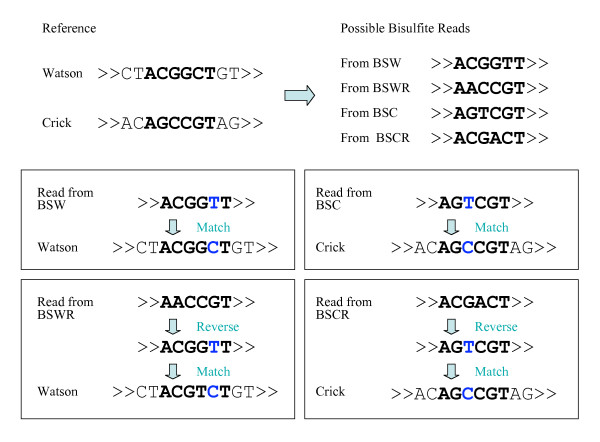
**Mapping bisulfite reads to 4 possible bisulfite strands (BSW/BSWR/BSC/BSCR) is equivalent to mapping the bisulfite read and its reverse complementary read to both Watson/Crick strands of the original reference sequence**.

As discussed earlier, each bisulfite read needs to be aligned to four bisulfite strands (BSW, BSWR, BSC, BSCR) instead of two normal strands (Watson and Crick) as in normal read mapping. In BSMAP, each bisulfite read and its reverse complementary sequence are aligned with both the Watson and the Crick strands of the reference sequence. This procedure is equivalent to mapping BS reads to four bisulfite strands (BSW, BSWR, BSC, and BSCR) (Figure 4).

BSMAP supports gapped/pair-end alignment and iterative trimming of low-quality base pairs, as well as parallel computing in multi-thread execution. In addition to providing unique hits, BSMAP can also report non-unique multiple hits with a user-defined maximum number of mismatches. Such "multiple hits" information may lead to more accurate estimates of the methylation ratio. In addition, BSMAP also provides the option of using a simple seeding HASH table, where all Cs are converted into Ts in the keys, as an alternative to the bisulfite seeding HASH table in mapping. This simple seeding option is slower but is more memory efficient and thus would be suitable for computers with less RAM.

### Algorithm comparisons

We compared BSMAP with CokusAlignment by mapping a real Solexa dataset containing 2,946,339 bisulfite reads (31 nt) to the *Arabidopsis *genome (119,707,899 nt) [4] (Table 2). BSMAP is about 6× faster than CokusAlignment with a similar mapping sensitivity. Overall, the mapping results for BSMAP and CokusAlignment were similar. It is worth noting that in BSMAP, up to 2 mismatches were allowed and the read was kept only if the second best hit had >= 1 more mismatches than the best hit; in CokusAlignment, the matching probability scores were calculated based on the original Solexa image file, and the score cutoff could not be explicitly associated with our BSMAP mapping criteria. Therefore, a strict comparison of the mapping sensitivities of BSMAP and CokusAlignment is not feasible.

Using the same *Arabidopsis *read data, we also simulated the other two bisulfite mapping approaches discussed earlier [2,3] and compared them with BSMAP. To simulate the RRBS bisulfite mapping used by Meissner et al. [2], all Cs were converted to Ts in the reads and references, and converted reads were aligned to the converted reference using SOAP. As discussed earlier, it was not feasible to record all non-unique mappings for post-processing, so reads with more than 100 mappings were discarded. The false-positive bisulfite C/T alignments were counted as mismatches, and only the resulting unique mappings (if there were any) were retained. Meissner mapping was found to detect fewer unique bisulfite mappings (39.1%) than BSMAP (44.9%) (Table 2). This is due to the fact that the false-positive bisulfite C/T alignments can introduce too many matches to some reads, which exceed the 100 mapping threshold and are therefore discarded. With a larger reference genome, more reads may be discarded because of the larger number of false-positive bisulfite C/T alignments, leading to even lower mapping sensitivity. The estimated speed of Meissner mapping is roughly half that of BSMAP. Because Meissner et al. used a custom-written mapping program, an exact comparison of speed is not applicable. We excluded the post-processing time in our estimation of speed, so the actual mapping speed would be slower than listed.

**Table 2:** Comparison of BSMAP and other mapping algorithms

	Speed reads/sec/CPU core)	Uniquely Aligned Reads (%)
BSMAP	146	44.9%
Cokus Alignment [4]	~25 [4]	45.1%
Meissner mapping [2]	83*	39.1%
Lister mapping [3]	125*	42.5%

* estimated speed based on SOAP, excluding the post processing time.

In simulating the mapping of Lister et al. [3], three reference sequences were used: the original *Arabidopsis *genome sequence, the Watson strand with all Cs converted to Ts, and the Crick strand with all Gs converted to As. For each read, the mapping results of the three references were merged and duplicated mappings were combined. The actual mismatches were recalculated by excluding the bisulfite alignments, and unique mappings were counted. As expected, Lister mapping reported fewer uniquely mapped reads than BSMAP (Table 2). It is worth noting that the sensitivity of Lister mapping highly depends on read length. Longer reads will contain more bisulfite C/T alignments and are therefore more likely to exceed the maximum mismatch threshold and become un-mappable. On the other hand, longer reads will have more true mismatches, such as SNPs, and thus will tolerate fewer bisulfite C/T alignments as mismatches. Therefore, with increased read length, we would expect a rapid drop in mapping sensitivity.

In summary, existing bisulfite mapping approaches either sacrifice mapping sensitivity for time or are too computationally expensive to be applied to large reference sequences. BSMAP offers efficient yet accurate bisulfite mappings through a fast bitwise masking algorithm. A great degree of complexity in bisulfite read mapping is introduced by the fact that within a single read there might be multiple CpGs with heterogeneous methylation status. By algorithm design, BSMAP is able to map multiple-CpG bisulfite reads to the genome considering every possible methylation pattern. BSMAP is also able to detect C methylation other than at CpG content [4], although it is difficult to determine whether the non-CpG C methylations are generated through a novel methylation mechanism or through incomplete bisulfite conversion.

Whole genome BS-seq is still too costly, especially for a comparative analysis of large mammalian genomes in multiple cell types and conditions. Recently, front-end array capture [8] coupled with bisulfite sequencing has become a popular cost-effective strategy to generate methylation profiling of a reduced representation of a large genome, e.g., promoters and CpG islands. Although the capture assay significantly enriches (usually by several hundred fold) the targeted sequences, only 50–90% (capture specificity) of the captured sequences are usually within the targeted regions. The remaining reads are from non-specific genome background. Therefore, we advise BS-seq users to still use the entire genome rather than reduced representation of the genome, as the searching space for bisulfite read mapping.

## Conclusion

We present a novel, flexible, and efficient general-purpose bisulfite sequence mapping program, BSMAP, for the analysis of whole genome shotgun BS-seq data. BSMAP uses the positions of all Cs in the reference sequences and applies bitwise masking to implement asymmetric C/T transition: T in bisulfite reads can be mapped to either C or T in the reference but not vice versa. The efficient seeding and HASH table used in BSMAP offer both improved flexibility and performance. Additionally, BSMAP supports the detection of multiple CpG heterogeneous methylation patterns and C methylations that are not at a CpG site. Finally, BSMAP is easy to use and supports gapped/pair-end alignment, iterative trimming of low-quality base pairs, and multi-thread parallel computing.

## Methods

### Dataset

BS-seq data from the *Arabidopsis *genome were obtained from [4]

### Software implementation

BSMAP was developed on Linux64 platform under GNU PL3 licenses, and the C++ source code is freely available at .

BSMAP takes standard FASTA/FASTQ format as input. The mapping output includes the following information: read ID, read sequence, quality score, read length, mapping flag (MA for matching, NM for not matching, OF for multiple matching overflow), mapping chromosome, position, strand, number of mappings with up to five mismatches, and detailed mismatch positions and nucleotide information in the read.

## Authors' contributions

YX and WL conceived the project and wrote the paper. YX developed the bisulfite seeding and bit masking algorithm, and coded the BSMAP software based on SOAP. YX and WL tested the BSMAP software. All authors read and approved the final manuscript.
